# Enhanced Predictive Accuracy of the Modified DECAF Score Over Neutrophil-Lymphocyte and Platelet-Lymphocyte Ratios in Chronic Obstructive Pulmonary Disease Exacerbations: A Comparative Study of Mortality and Hospital Stay Duration

**DOI:** 10.7759/cureus.79285

**Published:** 2025-02-19

**Authors:** Ashika N Adinarayan, Ajit Harsha, Srikanth Katare, Alamelu Haran, Mamatha S

**Affiliations:** 1 Respiratory Medicine, Vydehi Institute of Medical Sciences and Research Centre, Bengaluru, IND; 2 Respiratory Medicine, ESIC Medical College and PGIMSR and Model Hospital, Bengaluru, IND

**Keywords:** copd chronic obstructive pulmonary disease, copd exacerbation, decaf score, nlr, plr

## Abstract

Background: Chronic obstructive pulmonary disease (COPD) exacerbations contribute significantly to increased morbidity, mortality, and healthcare costs, necessitating effective predictive tools for patient outcomes. The modified DECAF score (baseline dyspnea, eosinopenia, consolidation, acidemia, and frequency of hospitalization in the last year), neutrophil-to-lymphocyte ratio (NLR), and platelet-to-lymphocyte ratio (PLR) are commonly evaluated for their predictive accuracy in COPD exacerbations.

Objective: This study aimed to compare the predictive accuracy of the modified DECAF score, NLR, and PLR in determining mortality and duration of hospital stay in patients with COPD exacerbations.

Methods: A prospective cohort study was conducted on 100 patients hospitalized with COPD exacerbations. Data on demographics, clinical history, and laboratory parameters were collected. The predictive performance of the modified DECAF score, NLR, and PLR was assessed using statistical analyses, including sensitivity, specificity, and multivariate regression.

Results: The modified DECAF score was significantly correlated with mortality (p < 0.01) and demonstrated superior predictive accuracy compared to NLR and PLR. While longer hospital stays were associated with higher modified DECAF scores, the correlations with NLR and PLR were not statistically significant. Environmental risk factors did not significantly impact mortality or the modified DECAF score.

Conclusion: The modified DECAF score is a more reliable predictor of mortality and hospital stay duration in COPD exacerbations than NLR and PLR. Its implementation in clinical practice could enhance patient management by enabling better risk stratification. Further research is needed to validate these findings across diverse populations and explore the integration of genetic and epigenetic markers.

## Introduction

Chronic obstructive pulmonary disease (COPD) exacerbations are acute events characterized by a significant worsening of a patient’s respiratory symptoms that necessitate additional therapeutic intervention [1]. These exacerbations are prevalent and pose substantial challenges in the management of COPD, a disease affecting millions worldwide. COPD exacerbations contribute to increased morbidity, frequent hospitalizations, and a significant economic burden on healthcare systems [2]. The clinical importance of COPD exacerbations lies in their association with adverse outcomes, including prolonged hospital stays, diminished quality of life, and heightened mortality rates. Understanding and predicting the outcomes of these exacerbations are essential for effective patient management and resource allocation [1]. COPD exacerbations can lead to numerous complications, such as acute respiratory failure, cardiovascular events, and increased susceptibility to infections. These complications often result in extended hospital stays and elevated healthcare costs. Moreover, exacerbations are associated with increased morbidity and mortality, highlighting the need for accurate predictive tools [1]. Traditionally, outcomes of COPD exacerbations are predicted using clinical assessments and composite scores that incorporate various patient factors. However, these methods often show variability in accuracy and reliability, necessitating the development of more standardized and objective predictive tools [3]. There is a critical need for predictive markers that can more accurately and objectively forecast patient outcomes during COPD exacerbations. Improved predictive accuracy can enhance clinical decision-making, optimize patient management, and better allocate healthcare resources.

The neutrophil-to-lymphocyte ratio (NLR), calculated by dividing the absolute neutrophil count by the absolute lymphocyte count, is an emerging marker for predicting various health outcomes. NLR is indicative of the systemic inflammatory response and has been associated with outcomes in respiratory diseases, particularly COPD exacerbations [4]. Similarly, the platelet-to-lymphocyte ratio (PLR), determined by dividing the platelet count by the lymphocyte count, serves as another inflammation indicator and has shown potential in predicting clinical outcomes in conditions, including COPD exacerbations [5]. The modified DECAF score is specifically tailored for COPD patients. It incorporates clinical and laboratory parameters to offer a comprehensive risk assessment for those hospitalized with exacerbations. It includes different parameters, such as baseline dyspnea, eosinopenia, consolidation, acidemia, and frequency of hospitalization in the last year. The score ranges from 0 to 6, with patients scoring 0-2 being considered as low risk, 3-5 as intermediate risk, and 4-6 as high risk. The modified score assigns different weights to parameters based on their predictive value in COPD patients, emphasizing arterial blood gases (PaO_2_ and PaCO_2_) over subjective measures such as dyspnea. It also incorporates common comorbid conditions in COPD patients, such as cardiovascular disease or diabetes. The importance of the modified DECAF score lies in its ability to provide a more accurate and tailored prognostic tool for COPD patients, helping clinicians predict the severity of exacerbations more accurately, identify high-risk patients needing intensive care, and optimize resource allocation and patient management, and it is a composite index designed to predict mortality in COPD patients [6].

Current literature presents varying degrees of support for the prognostic value of NLR, PLR, and the modified DECAF score. Studies have shown that elevated NLR and PLR are associated with worse outcomes in COPD exacerbations, yet inconsistencies and gaps remain regarding their comparative effectiveness. While the modified DECAF score has been validated in multiple cohorts, its performance relative to these novel hematologic ratios has not been thoroughly investigated. This study aims to address these gaps by conducting a comparative analysis of NLR, PLR, and the modified DECAF score in predicting mortality and the duration of hospital stay in patients with COPD exacerbations. By evaluating these predictive tools side by side, we seek to identify the most accurate and clinically useful marker for improving patient outcomes and resource management in COPD care.

The primary objectives of this study are to compare the predictive accuracy of NLR, PLR, and the modified DECAF score in determining mortality and the duration of hospital stay among patients experiencing COPD exacerbations. This comparison is critical for enhancing clinical decision-making in the management of COPD exacerbations. The findings of this study have the potential to inform clinical practices, leading to better patient care, optimized resource utilization, and improved patient outcomes.

## Materials and methods

The study was designed to compare the predictive accuracy of NLR, PLR, and the modified DECAF score in determining mortality and the duration of hospital stay among patients experiencing COPD exacerbations. Conducted as a prospective cohort study, it involved patients admitted to the hospital with a diagnosis of COPD exacerbation. Inclusion criteria were adults aged 18 and above, with a confirmed diagnosis of COPD and hospitalization due to COPD exacerbation. Patients with concurrent acute infections other than COPD exacerbation, hematologic disorders, recent blood transfusion, or incomplete medical records were excluded. The sample size was determined based on power calculations to ensure sufficient statistical power to detect differences between predictive tools.

Data collection included baseline demographic information, such as age, gender, and smoking history, as well as clinical histories such as the duration of COPD, comorbidities, and prior exacerbations. Clinical and laboratory parameters involved complete blood count (CBC) to obtain neutrophil, lymphocyte, and platelet counts for calculating NLR and PLR, blood gas analysis for arterial pH (for acidemia), chest radiograph results for the presence of consolidation, eosinophil count, and history of or current atrial fibrillation. Dyspnea was assessed using the modified Medical Research Council (mMRC) dyspnea scale.

Primary outcome measures focused on in-hospital mortality, while secondary outcomes included the duration of hospital stay, the need for intensive care unit (ICU) admission, and readmission within 30 days. Data were collected prospectively during the hospital stay and recorded in a standardized electronic database. The study received approval from the institutional ethics committee (ECR/747/INST/KA/2015/RR-21(VIEC)), and informed consent was obtained from all participants. Patient confidentiality was strictly maintained throughout the study. Statistical analysis compared the NLR, PLR, and modified DECAF scores. Additionally, sensitivity, specificity, positive predictive value (PPV), and negative predictive value (NPV) for each score were calculated. Multivariate regression analysis was performed to adjust for potential confounders.

The study's data availability was managed through the Vykohms Hospital Management System, with inclusion criteria requiring patients to be evaluated at the time of admission. Exclusion criteria included worsening treatment for an existing infection, known malignant disease, renal failure, hematological or rheumatological disease, or any additional diagnoses affecting lactate values. Initially, 167 patients with acute exacerbation of COPD were recruited. However, 18 patients declined treatment; 28 were diagnosed with other associated respiratory comorbidities such as bronchiectasis, pleural diseases, and diaphragm diseases; and 21 were discharged against medical advice, resulting in a final study population of 100 patients. Recorded data included gender, background chronic diseases, drug use, clinical follow-up, 28-day mortality, fever, pulse, blood pressure, respiratory rate, Glasgow Coma Scale (GCS) values, SpO_2_, eosinophil values, lactate, and pH.

The NLR was calculated as neutrophils divided by lymphocytes and the PLR as platelets divided by lymphocytes. The modified DECAF score, used to predict outcomes in patients with COPD, included parameters such as dyspnea (D), eosinopenia (E), consolidation (C), acidemia (A), and atrial fibrillation (F), with each parameter having a point value. The total score could range from 0 to 6 points.

The data analysis process involved entering information into Microsoft Excel (Microsoft® Corp., Redmond, WA) and analyzing it using SPSS = Statistical Product and Service Solutions (SPSS, version 22; IBM SPSS Statistics for Windows, Armonk, NY) and Medistica. pvalue.io, a graphic user interface to the R statistical analysis software for scientific medical publications (2019-2024). Categorical data were represented in frequencies and proportions, while continuous data were represented as means and standard deviations. The independent t-test was used to identify mean differences between two quantitative variables, and correlations were assessed with the Pearson correlation coefficient. Receiver operating characteristic (ROC) curves were constructed for NLR, PLR, and mortality, as well as for DECAF and mortality. A comparison of NLR and PLR with DECAF was conducted. Optimal cut-off points were chosen for calculating sensitivity, specificity, and positive and negative predictive values. Graphical representations of data were created using MS Excel, MS Word, and Medistica. pvalue.io, with a p-value of <0.05 considered statistically significant. The statistical software used included MS Excel, SPSS version 22, and Medistica. pvalue.io.

## Results

In the study, the 70-79 age group has the highest number of individuals, consisting of 18 males and seven females. Across all age groups, the number of females remains significantly lower than that of males. A sharp decline in both genders is observed in the 80-89 age group, where the male population drops from 18 to 6, and the female population decreases from 7 to 3. The youngest age group (30-39) has the lowest recorded population, with only one male and no females.

**Figure 1:**
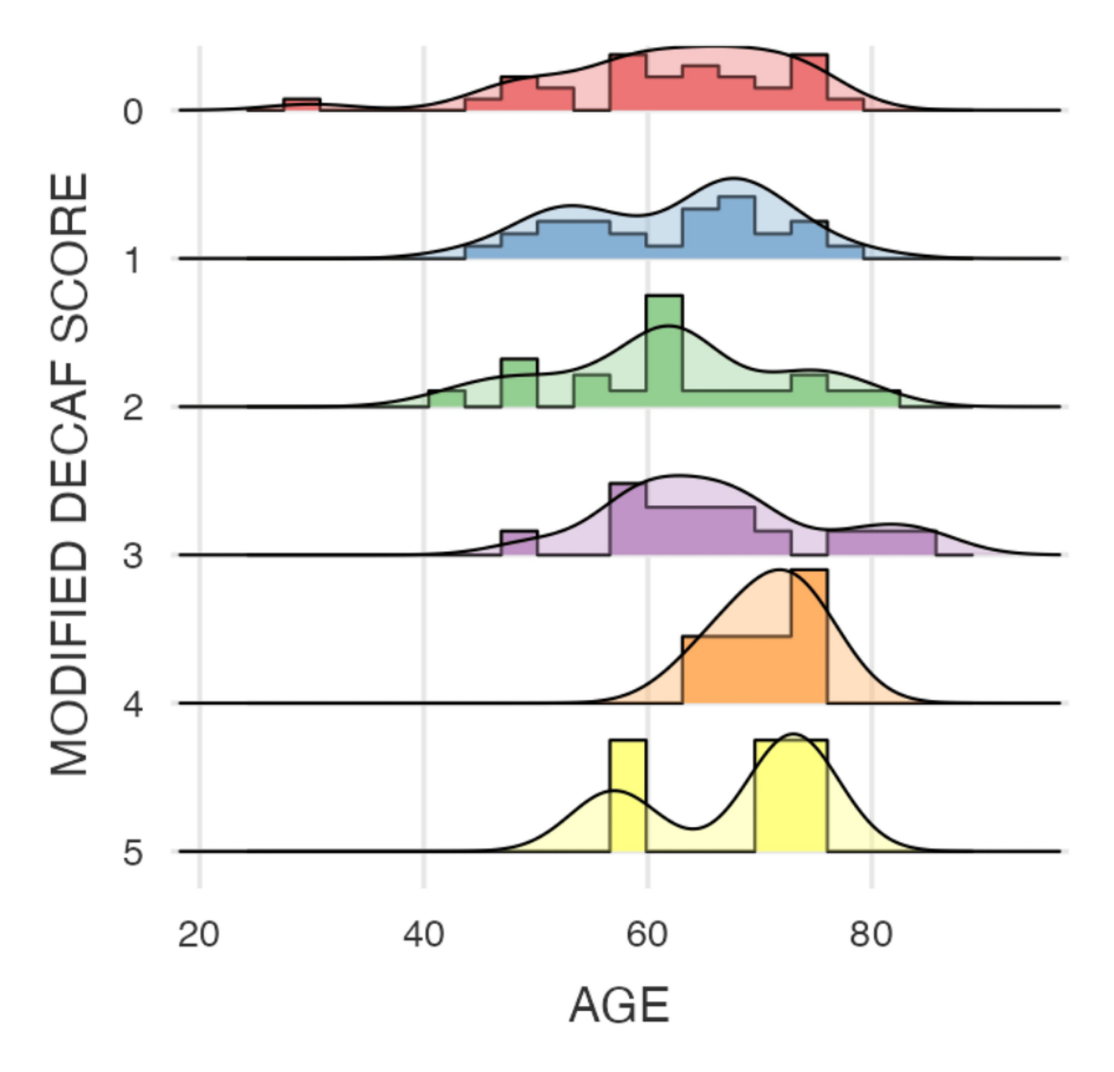
Histogram showing the distribution of modified DECAF scores among the patients 0–1 points → Low risk (lowest mortality risk, potential for early discharge) 2 points → Intermediate risk 3–5 points → High risk (increased mortality, consideration for intensive care)

**Figure 2:**
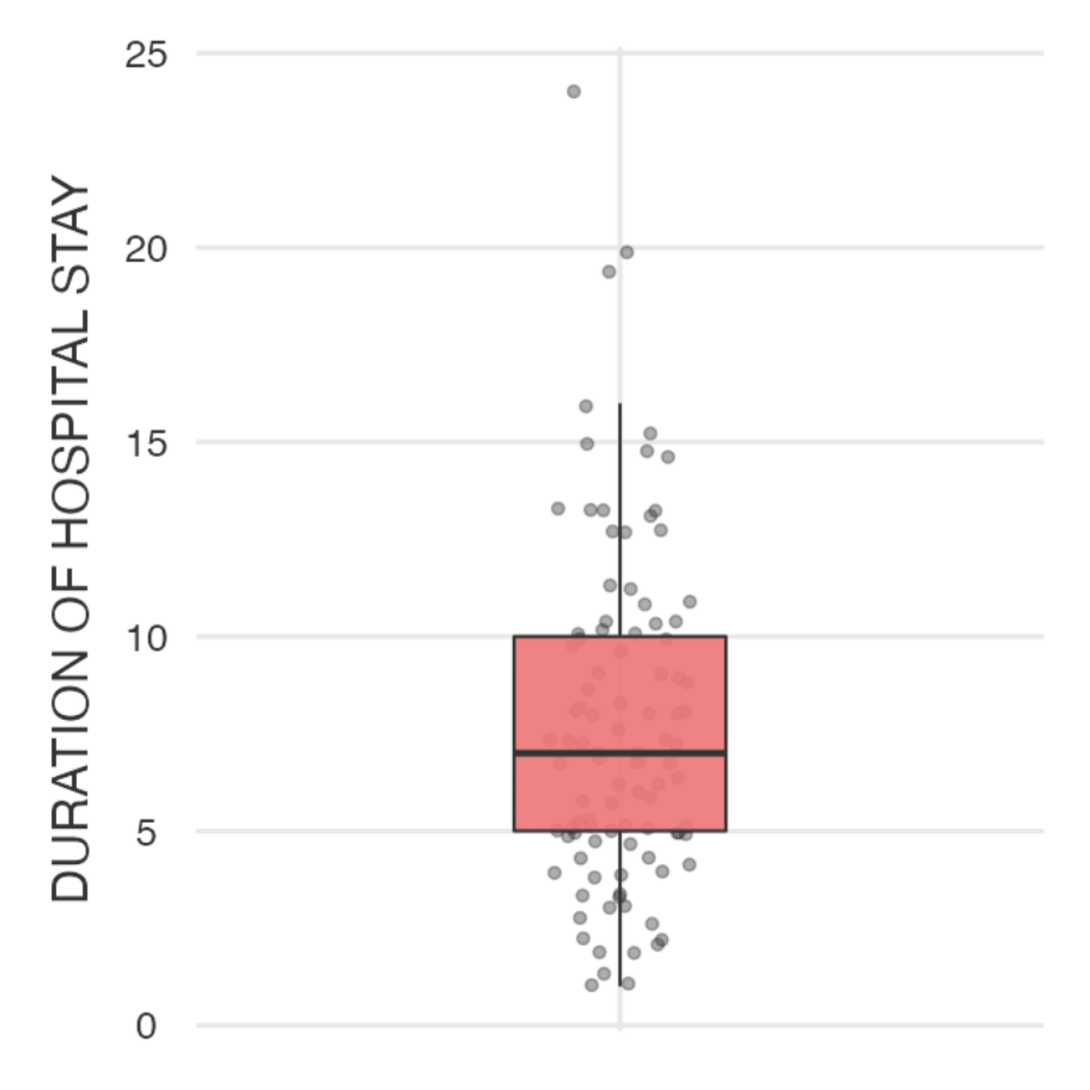
Box plot visualizing the spread and outliers in the duration of hospital stays

The descriptive statistics are presented in Tables 1-2 and Figure 1. The modified DECAF score had a mean value of 1.46, with a standard deviation of 1.34, indicating moderate variability among the observed scores. The median value was 1.00, with scores ranging from 0 to 5. This suggests that most patients had relatively low modified DECAF scores, though some cases exhibited higher values. The duration of hospital stay showed a mean of 7.64 days, with a standard deviation of 4.32 days. The median duration was 7.00 days, with hospital stays ranging from 1 to 24 days, as shown in Figure 2. The broad range suggests significant variability, potentially influenced by factors such as disease severity, treatment response, and comorbidities. Regarding inflammatory markers, the NLR had a mean of 1.80 and a standard deviation of 0.669, with a median of 1.72. The range varied from 0.736 to 2.97, indicating some degree of dispersion among the values, though the majority of cases remained within a narrow range. Similarly, the PLR exhibited a mean of 471, with a standard deviation of 106, a median of 462, and values ranging from 303 to 649. This suggests that, while there is some variability, most PLR values are clustered around the median.

**Table 1:** Univariable analysis by mortality Variable: N: Sample size P-value: P-value for statistical significance; Test: Statistical test used

Variable	Mortality (n=8)	No Mortality (n=92)	P-value	Test Used
Duration of hospital stay	10.0 (5.75; 13.0)	7.00 (5.00; 10.0)	0.37	Mann-Whitney
Modified DECAF score	4.00 (2.75; 4.00)	1.00 (0; 2.00)	<0.01	Mann-Whitney
Neutrophil-to-lymphocyte ratio (NLR)	1.82 (0.638)	1.80 (0.675)	0.93	Welch
Platelet-to-lymphocyte ratio (PLR)	471 (91.8)	471 (107)	0.99	Welch

**Table 2:** Key findings The variables being measured: Mean (SD): Mean and standard deviation; Median (Q25-Q75): Median and interquartile range; Min: Minimum value; Max: Maximum value; N: Sample size; Correlation coefficient (95% CI): Correlation coefficient and 95% confidence interval; P-value: P-value for statistical significance; Test: Statistical test used

Variable	Mean (SD)	Median (Q25-Q75)	Min	Max	n
Duration of hospital stay	7.64 (4.32)	7.00 (5.00; 10.0)	1.00	24.0	100
Modified DECAF score	1.46 (1.34)	1.00 (0; 2.00)	0	5.00	100
Neutrophil-to-lymphocyte ratio (NLR)	1.80 (0.669)	1.72 (1.28; 2.34)	0.736	2.97	100
Platelet-to-lymphocyte ratio (PLR)	471 (106)	462 (382; 560)	303	649	100

**Figure 3:**
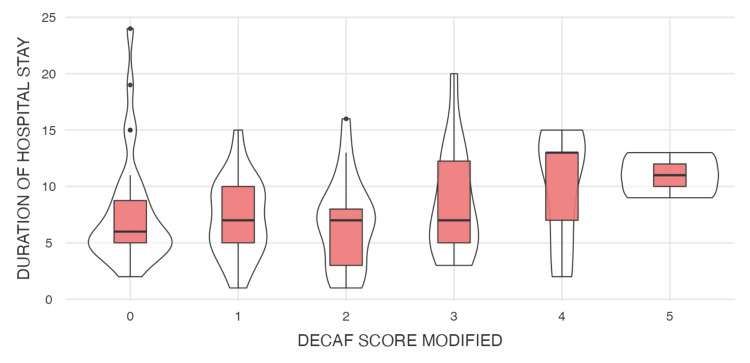
Box plot illustrating the relationship between the modified DECAF score and the duration of hospital stay

**Figure 4:**
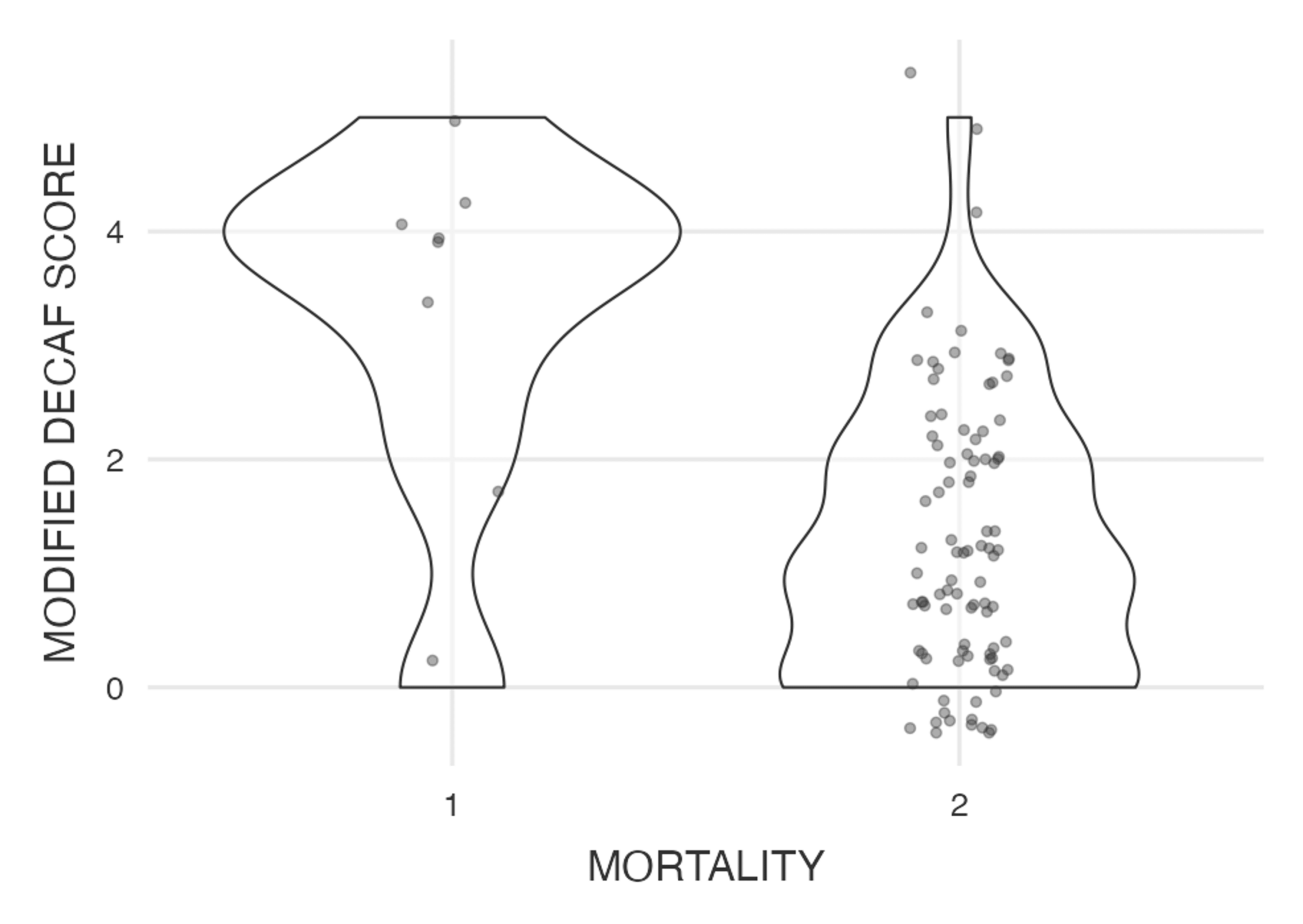
Violin plot of the modified DECAF score by mortality 1 - Mortality, 2 - No Mortality

**Figure 5:**
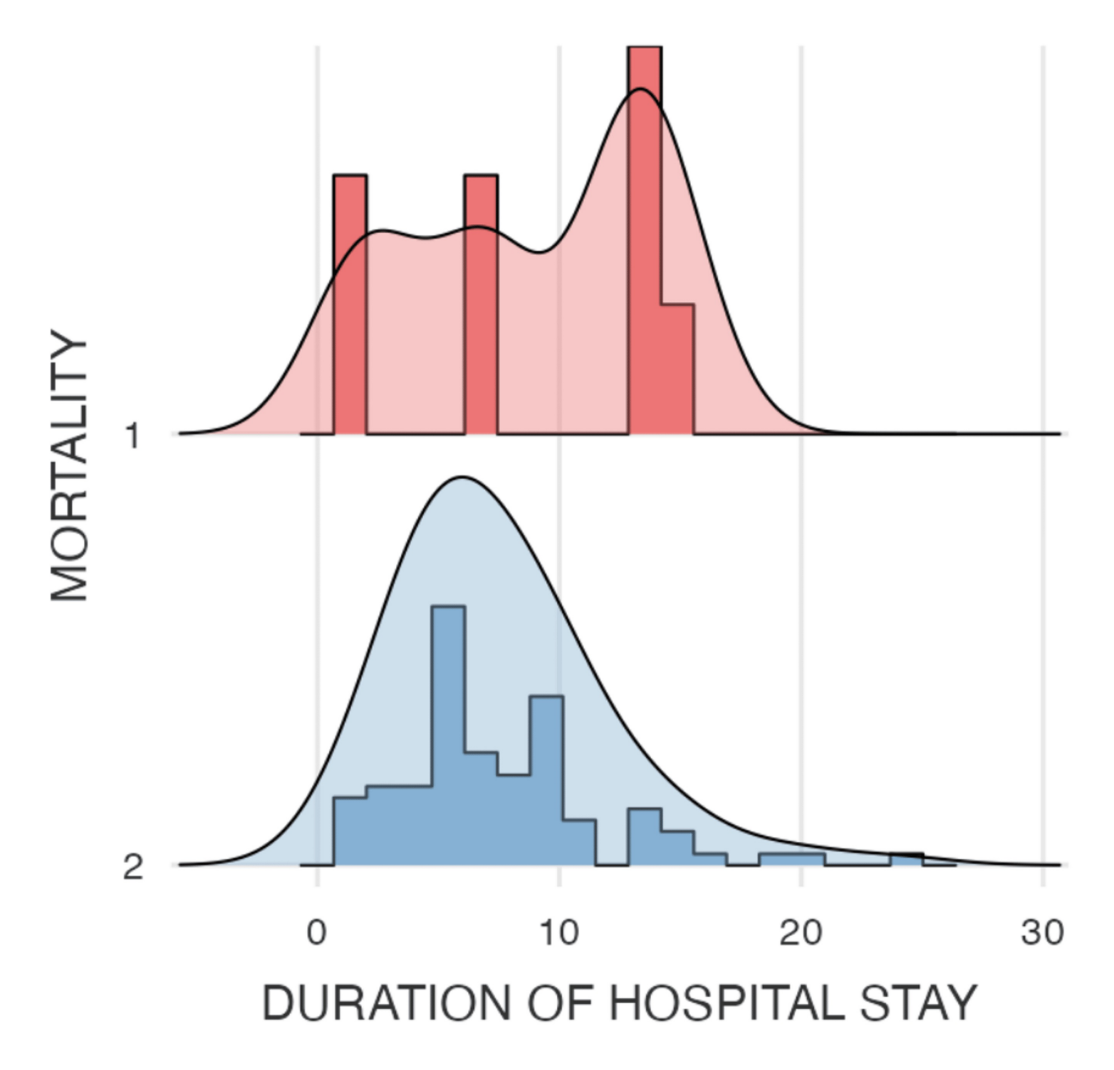
Density plot comparing the duration of hospital stays between the two mortality groups 1 - Mortality, 2 - No Mortality

**Figure 6:**
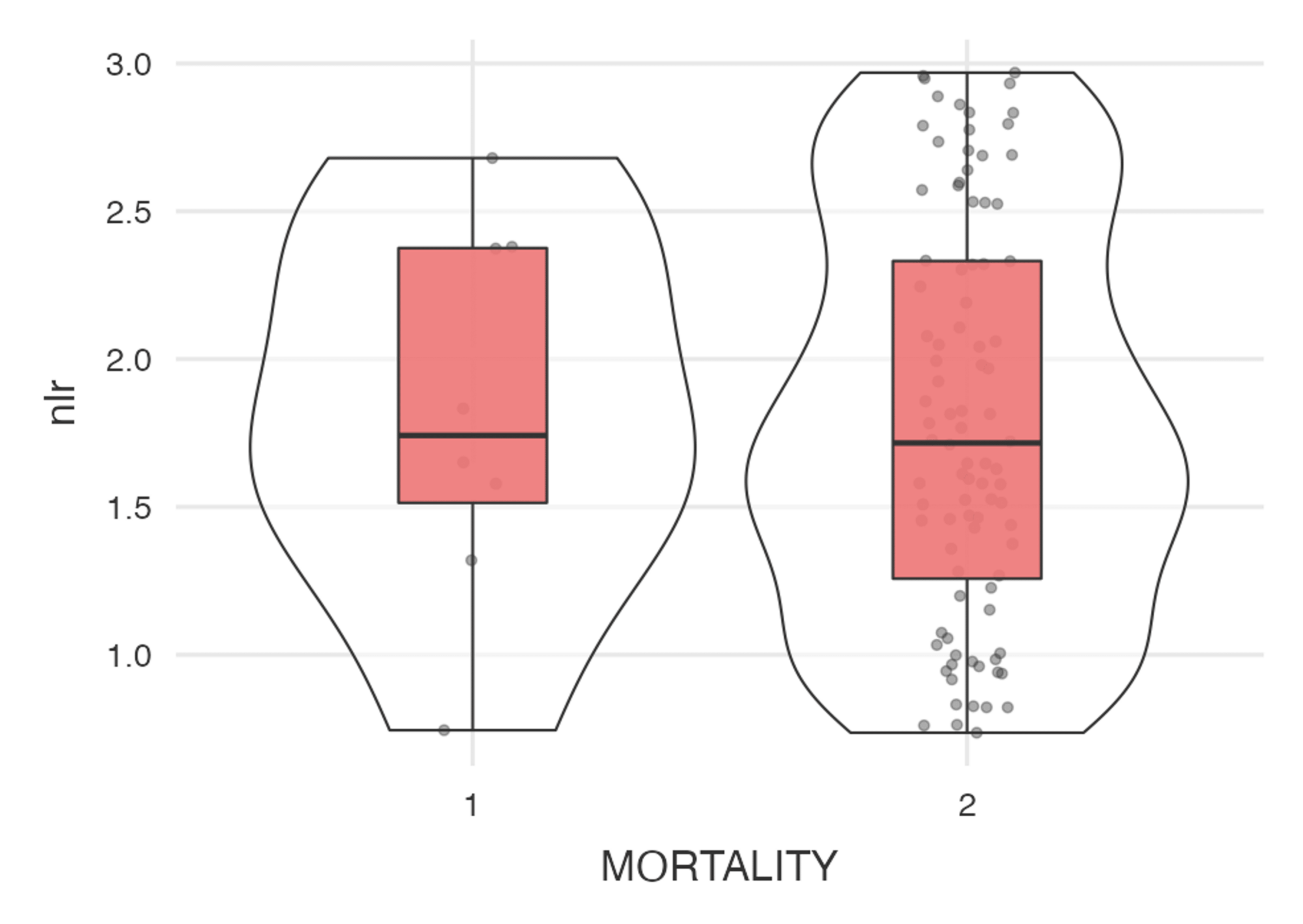
Violin plot comparing the meutrophil-lymphocyte ratio between the two mortality groups 1 - Mortality, 2 - No Mortality

**Figure 7:**
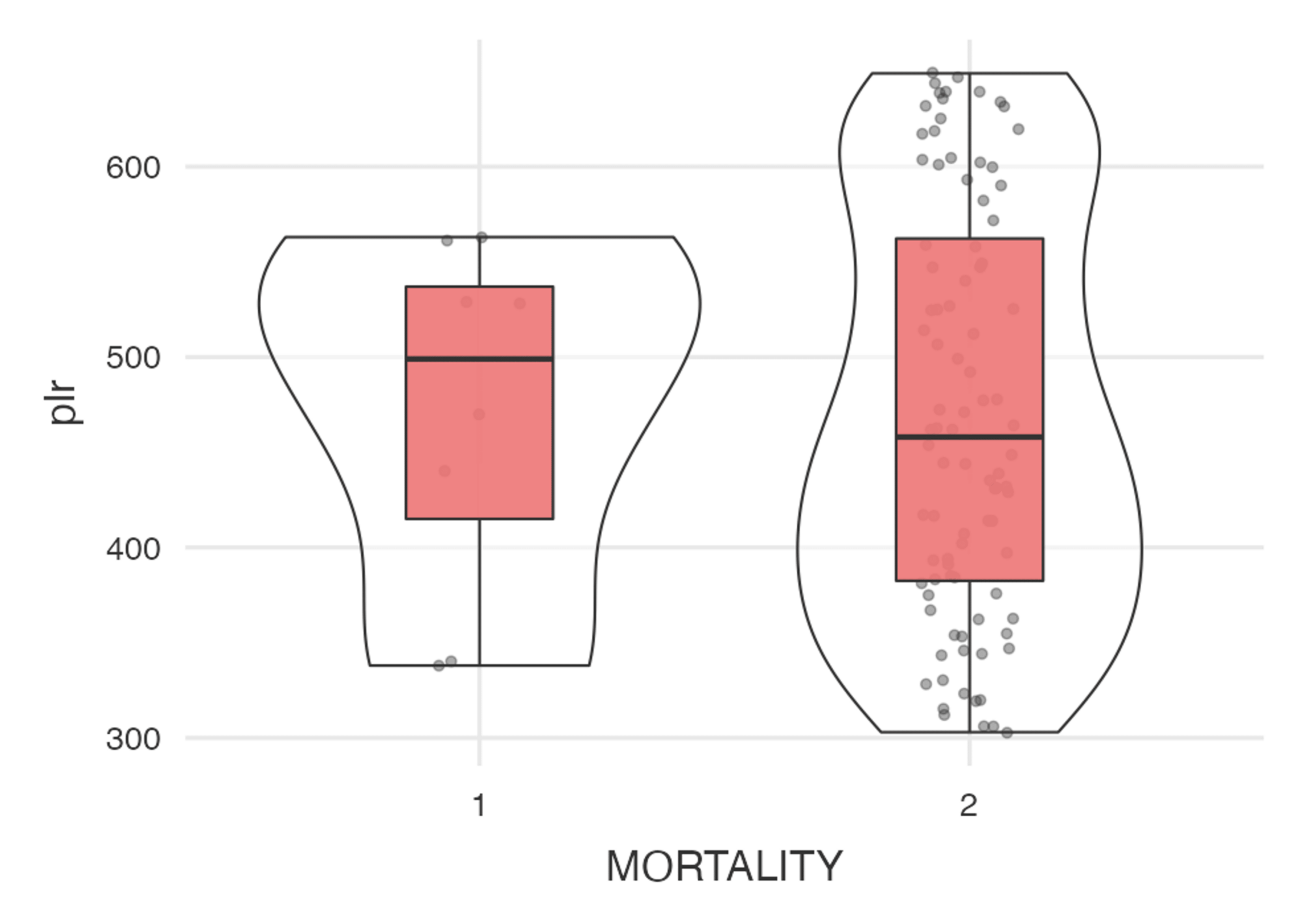
Violin plot comparing the platelet-lymphocyte ratio between the two mortality groups 1 - Mortality, 2 - No Mortality

The analysis summarized in Tables 1-2 shows the descriptive statistics and correlation analyses for the modified DECAF score, duration of hospital stay, NLR, and PLR. The modified DECAF score, as illustrated in Figure 4 (violin plot), shows a significant difference between the two mortality groups, indicating that higher DECAF scores are associated with increased mortality. Although patients with longer hospital stays (Figure 5) and higher DECAF scores tend to have worse outcomes, the correlation is not statistically significant, as shown in Figure 3 (scatter plot). NLR and PLR, as illustrated in Figures 6-7, respectively, do not show significant correlations with mortality or hospital stay duration. Environmental risk factors, as summarized in Table 1, do not significantly impact the modified DECAF score or mortality rates among the study cohort. Overall, the modified DECAF score is a better predictor of mortality compared to NLR and PLR. While longer hospital stays are associated with higher mortality, the lack of a statistically significant correlation suggests that other factors may influence patient outcomes.

## Discussion

The superior performance of the modified DECAF score suggests that it should be integrated into routine clinical practice for assessing patients with COPD exacerbations. Its comprehensive approach allows for a more nuanced understanding of patient risk, facilitating timely and targeted interventions. For instance, patients identified as high-risk could be prioritized for intensive monitoring, early initiation of non-invasive ventilation, and prompt escalation of care if necessary.

The burden of COPD exacerbations, as highlighted by Wedzicha et al. [1], continues to present significant health and economic challenges, characterized by increased morbidity, mortality, and healthcare costs. These exacerbations often lead to extended hospital stays, as observed in our study, where the mean duration was 7.64 days, with a maximum of 24 days. This not only imposes a financial burden on patients but also underscores the critical need for optimizing therapeutic strategies to reduce hospital admissions and mortality [1].

Our study further corroborates the value of the modified DECAF score as a predictive tool for mortality in COPD patients. The significant correlation between higher DECAF scores and mortality, with a p-value of <0.01, suggests that this score remains a reliable marker for identifying high-risk patients. Despite the clear association between longer hospital stays and higher mortality, as depicted in our analysis, the lack of statistical significance in this correlation hints at the complexity of factors influencing patient outcomes. This indicates that, while hospital duration is an important factor, other underlying conditions or variables may contribute to the overall prognosis, necessitating a broader approach to patient management.

Interestingly, the role of NLR as a potential biomarker for COPD prognosis, as discussed in Gao et al.'s 30-year longitudinal study, is not fully supported by our findings [4]. While NLR has been shown to correlate with lung function decline and COPD risk in other studies, our results did not demonstrate a statistically significant relationship between NLR and mortality. This discrepancy may be attributed to differences in study design, population characteristics, or other confounding factors that were not accounted for in our analysis. The range of NLR in our study (0.736-2.97) did not align with the expected outcomes based on previous research, suggesting that NLR’s utility as a prognostic marker may be context-dependent and possibly influenced by other clinical variables.

The exploration of DNA methylation in relation to NLR and lung function, as presented in the literature, offers a novel perspective on the potential epigenetic mechanisms underlying COPD progression. The association between higher NLR and reduced methylation at specific gene sites, such as cg05575921, proposes that inflammation, reflected by elevated NLR, might induce epigenetic modifications that contribute to lung function decline. While our study did not directly assess DNA methylation, the integration of such biomarkers in future research could provide deeper insights into the molecular pathways involved in COPD and enhance the predictive power of clinical tools such as the modified DECAF score [4-6].

The practical implications of our findings suggest that, while the modified DECAF score remains a superior predictor of mortality in COPD exacerbations, the role of NLR as a biomarker requires further investigation. The absence of a significant correlation between NLR, PLR, and mortality in our cohort implies that these markers may not universally apply across all patient populations. Thus, personalized approaches to COPD management, taking into account a range of clinical indicators, are essential for optimizing patient outcomes.

In conclusion, our study reinforces the importance of the modified DECAF score in predicting mortality in COPD patients while highlighting the need for a cautious interpretation of NLR and PLR as prognostic markers. The economic burden associated with extended hospital stays, coupled with the mortality risk in high DECAF score patients, underscores the urgency of refining therapeutic strategies. Future research should focus on integrating genetic and epigenetic data to uncover the complex interactions between inflammation, gene expression, and COPD progression, ultimately leading to more tailored and effective clinical interventions.

Limitations

Despite its strengths, this study has several limitations. The sample size, although diverse, may not be representative of all COPD populations, potentially limiting the generalizability of the findings. Additionally, the retrospective nature of the study may introduce bias in data collection and analysis. Future prospective studies with larger cohorts are needed to validate these findings and further refine the predictive models.

Future directions

Future research should focus on validating the modified DECAF score across different healthcare settings and populations. Additionally, exploring the integration of other biomarkers and clinical parameters could enhance the predictive accuracy of existing scores. Personalized medicine approaches, incorporating genetic and molecular profiling, may also offer new insights into risk stratification and management of COPD exacerbations.

## Conclusions

In conclusion, the modified DECAF score (scores ranging from 0 to 6, helping categorize patients based on their risk level: low risk (0-2 points) - patients are at a lower risk of severe exacerbations; intermediate risk (3-5 points) - patients have a moderate risk and may require closer monitoring; and high risk (4-6 points) - patients are at a higher risk of complications and may need intensive care) is a superior predictor of mortality and duration of hospital stay in patients with COPD exacerbations compared to NLR and PLR. Its implementation in clinical practice could significantly improve patient outcomes through better risk stratification and targeted management. Continued research is essential to confirm these findings and further enhance the tools available for managing COPD exacerbations.
